# Inclusion of a care bundle for fever, hyperglycaemia and swallow management in a National Audit for acute stroke: evidence of upscale and spread

**DOI:** 10.1186/s13012-019-0934-y

**Published:** 2019-09-02

**Authors:** Tara Purvis, Sandy Middleton, Louise E. Craig, Monique F. Kilkenny, Simeon Dale, Kelvin Hill, Catherine D’Este, Dominique A. Cadilhac

**Affiliations:** 1 Stroke and Ageing Research, School of Clinical Sciences at Monash Health, Monash University, Level 3, Hudson Institute Building, 27-31 Wright Street, Clayton, Victoria 3168 Australia; 2 Nursing Research Institute, St Vincent’s Health Australia Sydney, St Vincent’s Hospital Melbourne, Sydney, New South Wales Australia; 3 School of Nursing, Midwifery and Paramedicine, Australian Catholic University, Sydney, New South Wales Australia; 4 Centre for Research in Evidence-Based Practice, Bond University, Robina, Queensland Australia; 5 Stroke Division, The Florey Institute of Neuroscience and Mental Health, Heidelberg, Victoria Australia; 6 Stroke Foundation, Melbourne, Victoria Australia; 7 National Centre for Epidemiology and Population Health, Research School of Population Health, ANU College of Health and Medicine, Canberra, Australian Capital Territory Australia; 8 School of Medicine and Public Health, University of Newcastle, Sydney, New South Wales Australia

**Keywords:** Stroke, Health services research, Quality improvement, Audit and feedback, Care bundles

## Abstract

**Background:**

In the Quality in Acute Stroke Care (QASC) trial undertaken in stroke units (SUs) located in New South Wales (NSW), Australia (2005–2010), facilitated implementation of a nurse-led care bundle to manage fever, hyperglycaemia and swallowing (FeSS protocols) reduced death and disability for patients with stroke. We aimed to determine subsequent adherence to the bundled FeSS processes (reflective of the protocols) between 2013 and 2017 in Australian hospitals, and examine whether changes in adherence to these processes varied based on previous participation in the QASC trial or subsequent statewide scale-up (QASCIP—Quality in Acute Stroke Care Implementation Project) and presence of an SU.

**Methods:**

Cross-sectional, observational study using self-reported organisational survey and retrospective clinical audit data from the National Acute Services Stroke Audit (2013, 2015, 2017). Mixed-effects logistic regression was performed with dependent variables: (1) composite outcome measure reflecting compliance with the FeSS protocols and (2) individual FeSS processes, including the year of audit as an independent variable, adjusted for correlation of outcomes within hospital. Separate models including interaction terms between the year of audit and previous participation in QASC/QASCIP and year of audit and SU were also generated.

**Results:**

Hospital participation included the following: 2013—124 hospitals, 3741 cases; 2015—112 hospitals, 4087 cases; and 2017—117 hospitals, 4192 cases. An 80% increase in the odds of receiving the composite outcome in 2017 compared to 2013 was found (2013, 30%; 2017, 41%; OR 1.8; 95% CI 1.6, 2.0; *p* < 0.001). The odds of FeSS adherence from 2013 to 2017 was greater for hospitals that had participated in QASC/QASCIP relative to those that had not (participated OR 2.1; 95% CI 1.7, 2.7; not participated OR 1.6; 95% CI 1.4, 1.8; *p* = 0.03). Similar uptake in adherence was evident in hospitals with and without an SU between 2013 and 2017.

**Conclusion:**

The use of the FeSS protocols within Australia increased from 2013 to 2017 with the inclusion of these care processes in the National Audit. Greater uptake in hospitals previously involved in QASC/QASCIP was evident. Our implementation methods may be useful for other national initiatives for improving access to evidence-based practice.

**Electronic supplementary material:**

The online version of this article (10.1186/s13012-019-0934-y) contains supplementary material, which is available to authorized users.

## Background

Similar to other areas of healthcare, the need to reduce the evidence-practice gap remains a challenge in stroke management. Within Australian hospitals, there is a marked variation in the quality of stroke care provided [1, 2]. For example, access to stroke units ranges from 0–98% and the provision of thrombolysis from 0–20% [2], despite both of these processes being recommended in national guidelines [3]. It is well recognised that there are a number of challenges to embedding evidence into routine clinical practice. Translating research knowledge into practice requires clinicians to change behaviour [4], which can be a difficult and lengthy process, and is usually influenced by a wide range of factors [5]. No single approach is successful for all healthcare settings. Strategies that have taken into account contextual issues and barriers and facilitators to their implementation have had success in changing clinician behaviours and closing the evidence-practice gap [4–7].

Very few evidence-based nursing interventions exist for stroke. The Quality in Acute Stroke Care (QASC) trial was a complex healthcare intervention, involving multi-disciplinary supported nurse-initiated protocols for monitoring and managing of fever, hyperglycaemia (high sugar) and swallow dysfunction (FeSS protocols) post-stroke. This cluster randomised controlled trial was undertaken in 19 stroke units in New South Wales (NSW), Australia, throughout 2005–2010 [8]. The intervention consisted of barrier identification, multidisciplinary teamwork, educational outreach, local adaption, use of site champions and reminders [8]. An improvement in the quality of care provided [9], a 16% reduction in death and disability 90 days following stroke [8] and potential long-term survival benefits were shown [10]. Following on, the FeSS protocols were systematically introduced with training and the other strategies into all 36 stroke services (31 with a stroke unit) within one Australian state, NSW. The Quality in Acute Stroke Care Implementation Project (QASCIP) was conducted during 2013–2014, to evaluate the implementation strategies used in the original trial to promote ‘scale up and spread’ of this proven intervention. Improvements in adherence to the three FeSS protocols were found, demonstrating the successful statewide scale-up of this complex, quality-improvement intervention [6].

With the positive outcomes, individual indicators reflecting the bundled care processes in the FeSS protocols were included in the voluntary, biennial, National Stroke Audit programme (Australia) in 2013. This allowed the individual care processes, not already part of the audit (e.g. fever and glycaemia processes) to be monitored, nationally. No further national, systematic effort was made to implement the specific QASC intervention. However, uptake of the FeSS protocols may have been indirectly supported via this audit and feedback process, which was not part of the original QASC/QASCIP intervention. Moreover, the data from the National Audit were not specifically bundled as ‘FeSS processes’ in reports back to hospitals, which included a summary of some of these indicators.

Implementation of care bundles, which comprise of a small number of evidence-based interventions, that when implemented together, improve patient outcomes [11], have been used to improve care in a variety of health areas including chronic obstructive pulmonary disease, ventilator-associated pneumonia [12], catheter-related blood stream infections [13], and delirium [14]. Inclusion of these bundles in national audits [15] and registries [16, 17] have been used to effect.

The level of translation of the FeSS protocols into standard stroke care across Australia, beyond the hospitals involved in QASC and QASCIP, is unknown. The aim of this study was to determine adherence to specific FeSS processes of care (reflective of the protocols) between 2013 and 2017 in Australian hospitals delivering acute stroke care as a case study to highlight evidence translation in stroke. Secondary aims included establishing whether changes in adherence over time varied by participation in the QASC trial or QASCIP and in hospitals with and without a stroke unit (SU). We also wanted to compare adherence to the individual FeSS processes in the 2017 National Audit to results of QASC and QASCIP.

## Methods

Cross-sectional data were used in this observational study, from hospitals participating in the biennial National Stroke Audit–Acute Services programme (Australia) during 2013, 2015 and 2017 [18]. The programme entails a self-reported organisational survey and a retrospective clinical medical record audit. The survey captures information on organisational features of the service comprising bed numbers, service characteristics including the use of management protocols for fever and available resources. The clinical medical record audit is performed by trained data abstractors from each hospital. The first 40 consecutive case records of patients with a primary diagnosis of stroke (ICD-10 codes: I61, I62.9, I63, I64) are audited from admissions over a 6-month period starting in June (the previous year). For example, data included in the 2013 audit reflected patient admissions from 2012. Data are captured on a specifically designed web tool using standardised procedures. Patient demographic characteristics, adherence to processes of care, and in-hospital outcomes are collected [19]. Up to five cases from hospitals are re-audited by a second abstractor to determine the inter-rater reliability.

### Data collection

**Fig. 1:**
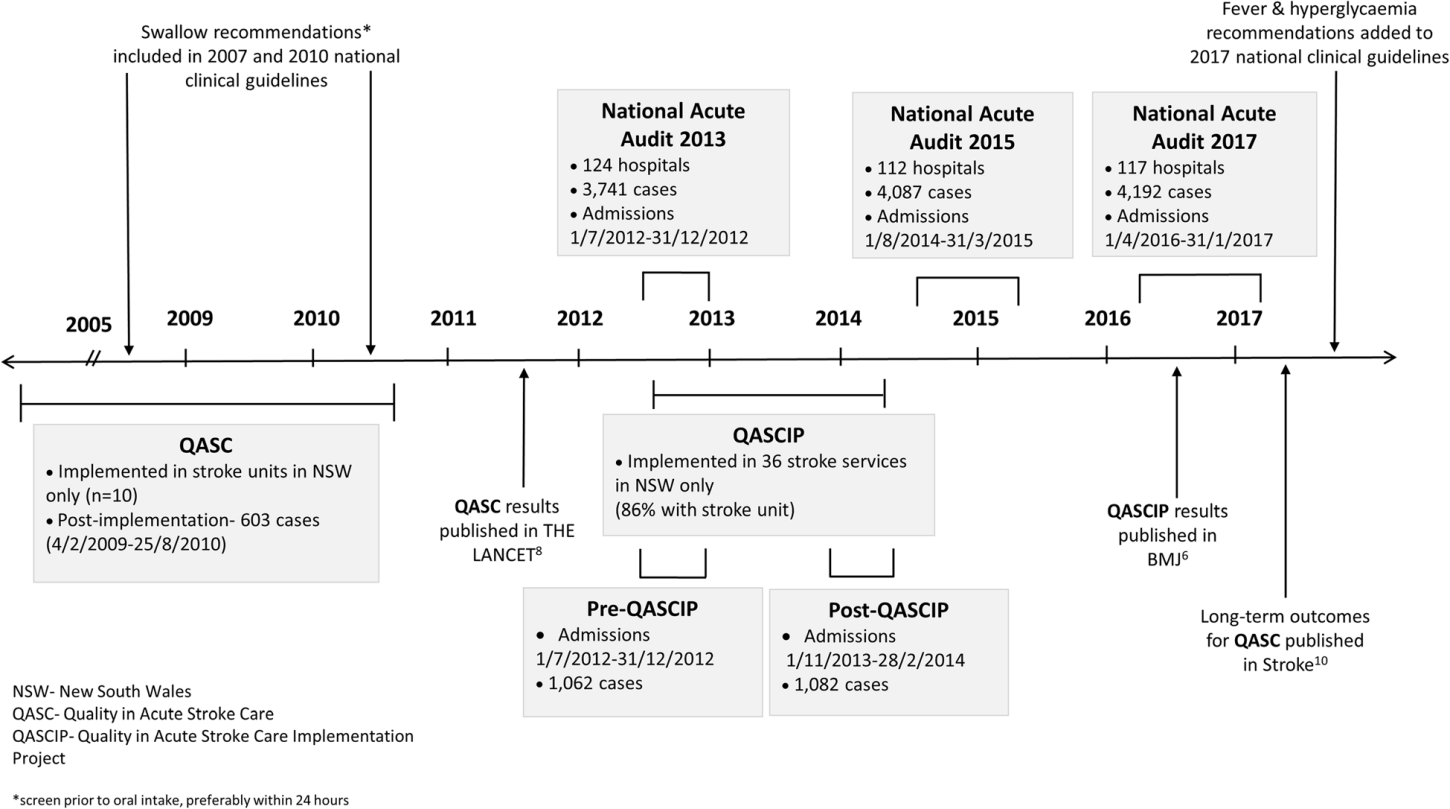
Timeline of data collection and publications for QASC, QASCIP and National Audits

Nine indicators of adherence to the FeSS protocols were developed and included in the Stroke Foundation audit programme in 2013 after the publication of the positive results of the QASC trial (herein referred to as FeSS processes). These were also collected in QASCIP. Other than processes relating to swallow function (prior to oral intake), no other indicators related to the FeSS processes were included in the National Audit prior to 2013. After the 2013 audit, changes were made by the Stroke Foundation to reduce data burden and align questions with the current Acute Stroke Services Framework [20] and the Acute Stroke Clinical Care Standards [21]. Thus, not all processes from the FeSS protocols collected in the 2013 audit were collected in 2015/2017. Three processes not collected related to the monitoring of fever and hyperglycaemia in the first 3 days of the admission, with all treatment-related processes remaining consistent (Table 1). Figure 1 displays the timeline of data collection and publications for QASC, QASCIP and the National Audits.

**Table 1:** Comparison of processes collected in QASC, QASCIP and National Audits, with outline of FeSS processes included for analyses

	Processes included in the FeSS protocols	QASC	QASCIP	National Audits			Processes included in the current study and definition foranalysis
				2013	2015	2017	
	Fever						
	Temperature recorded at least four times on day 1, day 2 and day 3^a^	✓	✓	✓	×	×	
	Received paracetamol within 1 h of the first febrile event (recorded in first 72 h)	✓	✓	✓	✓	✓	✓
F1	Overall fever treatment						Patients without fever classified as receiving appropriate fever treatment
	Hyperglycaemia						
	Formal venous blood glucose level recorded in the ED	✓	✓	✓	×	×	
	Finger prick glucose recorded at least four times on day 1, day 2 and day 3^a^	✓	✓	✓	×	×	
	Insulin within 1 h if glucose > 10 mmol/L^b^ (recorded in the first 72 h)	✓	✓	✓	✓	✓	✓
G1	Overall hyperglycaemia treatment						Patients without high glucose classified as receiving appropriate hyperglycaemia treatment
	Swallow						
S1	Swallow screen or swallow assessment by speech pathologist within 24 h of admission	✓	✓	✓	✓	✓	✓
S2	Swallow screen or assessment before food or drink	✓	✓	✓	✓	✓	✓
S3	Swallow screen or assessment before oral medications	✓	✓	✓	✓	✓	✓
S4	Overall swallow monitoring						Met all of S1, S2 and S3 (total cohort)
S5	Swallow assessment by speech pathologist if failed swallow screen	✓	✓	✓	✓	✓	✓ Those who did not fail swallow screen or did not receive the screen were classified as receiving appropriate swallow treatment
S6	Overall swallow monitoring and treatment						If monitoring elements S1, S2, S3 and treatment element S5 all met
	Composite outcome All fever, sugar and swallow elements						Met all of F1, G1 and S6

*FeSS* fever, sugar swallow; *QASC* Quality in Acute Stroke; *QASCIP* Quality in Acute Stroke Implementation Project; *ED* emergency department

^a^Question was asked individually for each of day 1, day 2 and day 3

^b^Finger prick glucose greater than 10 mmol/L (in QASC used 11 mmol/L, with changes reflecting recent updates in Australian guidelines)

### Statistical analysis

Data from hospitals participating in any audit were included in analyses. ‘Not documented’ and ‘unknown’ responses to the categorical questions related to adherence were assumed to be negative and included in the denominator. Only the six FeSS processes common in all three audits were included. As with the earlier trial, the process of care outcome was a derived composite measure, which reflected compliance to all fever, hyperglycaemia and swallow processes (see Table 1). Decision rules around the composite outcome were consistent with QASCIP, where applicable [6]. Adherence to the individual FeSS processes was reported in order to assess uptake into clinical practice of specific components. Characteristics of patients and hospitals were compared across the three audits using the chi-square test for categorical variables and the Kruskal-Wallis test for continuous variables.

To assess whether adherence to the FeSS processes changed over time, and if so, between which years, separate logistic regression models, which included the year of the audit, were generated for the composite outcome and each individual process. A mixed-effects model was used to adjust for potential correlation of outcomes within hospitals.

For the composite outcome, we also generated additional models which included an interaction term between year of audit and (a) previous participation in QASC or QASCIP and (b) presence of an SU, to determine if adherence to the FeSS processes over time differed by previous involvement in QASC/QASCIP or presence of a stroke unit. The *p* values for the interaction term were used to determine whether the factor was associated with a change in adherence. We did not adjust for patient characteristics in our primary analyses since the composite outcome was relevant to all patients, and it is assumed that all eligible patients should receive the recommended care processes regardless of stroke severity or other factors. We also performed sensitivity analyses where patient characteristics and organisational factors with *p* < 0.1 on univariable analyses and variables with clinical importance (such as age and sex) were included in the modelling for adherence to the individual processes and the composite outcome.

The level of significance for all statistical tests was *p* < 0.05. Adjusted odds ratios and 95% confidence intervals (CIs) are presented for all multivariable results. Data were analysed using Stata SE 15.0 [22].

Post hoc power calculations provided evidence that the study had at least 80% power to detect absolute differences in adherence to the composite outcome between audit years of 4.5–6.5%, with a 5% significance level. This calculation assumed an average of 30–36 audits per hospital, intraclass correlation coefficients of 0.02 to 0.1, consistent with previous work [8, 23], resulting in design effects of 2–4 due to the correlation of outcomes within hospitals [24].

## Results

A total of 124 hospitals provided 3741 cases to the 2013 audit, with 112 hospitals involved in 2015 (4087 cases), and in 2017, 4192 cases were provided by 117 hospitals. In 2013, 17 hospitals providing data to the audit had participated in QASC, contributing 620 cases (17%). In 2015, 32 hospitals had either participated in QASC or QASCIP (1158 cases, 28%), and in 2017, this included 35 hospitals (1280 cases, 31%).

The majority of participating hospitals in the clinical audits had an SU (2013, 87; 2015, 88; 2017, 94). However, a total of 1930 patients were still treated in a hospital without an SU in the audits (2013, 673; 2015, 664; 2017, 593).

The other organisational characteristics of hospitals participating in each audit are presented in Table 2. There was no difference in the proportion of participating hospitals that had an SU, a stroke clinical care pathway or regular stroke multidisciplinary team meetings across the three audit periods. Statistically significant increases over time were evident in the proportion of hospitals reporting the use of protocols for fever (2013 77%, 2017 92%; *p* < 0.001), hyperglycaemia (2013 81%, 2017 91%; *p* = 0.009) and swallow dysfunction (2013 85%, 2017 97%; *p* = 0.001). The median age of participants across all cohorts was 76 years, 55% were male, and 25% had a history of diabetes. The proportion of patients presenting with an ischaemic stroke varied significantly across time; however, differences were small (2013 77%, 2015 76%, 2017 80%; *p* < 0.001). There was a reduction in the severity of stroke across the audits, as indicated by differences in the proportion with arm weakness, and an inability to walk on admission, and incontinence within 72 h reported from the 2013 to 2017 audits (please see Additional file 1).

**Table 2:** Organisational characteristics of hospitals participating in the National Audits

Organisational characteristics of hospitals participating in the clinical audit	2013 Audit,*N* = 124	2015 Audit,*N* = 112	2017 Audit,*N* = 117	*p* value^a^
Dedicated stroke unit	87 (70%)	88 (79%)	94 (80%)	0.14
Clinical care pathway for managing stroke	94 (76%)	93 (83%)	99 (85%)	0.18
Regular stroke multidisciplinary team meetings	115 (93%)	106 (95%)	107 (91%)	0.64
Agreed management (including assessment/monitoring) protocols for:				
Fever	95 (77%)	103 (92%)	108 (92%)	< 0.001
Hyperglycaemia	100 (81%)	104 (93%)	106 (91%)	0.009
Swallow dysfunction	106 (85%)	107 (96%)	114 (97%)	0.001

^a^From chi-square test

The proportion of patients with reported fever and high glucose levels in the first 48 h of admission decreased significantly over time, although changes were small (fever—2013 15%, 2015 12%, 2017 11%, *p* < 0.001; high glucose—2013 19%, 2015 17%, 2017 16%, *p* = 0.003) (data not shown). Compared with 2013 (30%), adherence to the composite outcome improved in 2017 (41%) (Table 3). With multivariable analyses, we found an 80% overall increase in the odds of receiving all FeSS processes (composite outcome) in 2017 compared to 2013 (OR 1.8; 95% CI 1.6, 2.0; *p* < 0.001), with a small change between 2013 and 2015. Adherence to individual fever and hyperglycaemia processes and most individual swallow components (except for swallow treatment) improved from 2013 to 2017, with greater improvements evident between 2015 and 2017, compared to 2013–2015 (Table 3).

**Table 3:** Adherence to composite outcome and individual FeSS processes (2013–2017)

FeSS monitoring and treatment processes	Univariable			Multivariable		
	2013,*N* = 3741,*n* (%)	2015,*N* = 4087,*n* (%)	2017,*N* = 4192,*n* (%)	2013^a^–2015,OR (95% CI)	2013^a^–2017,OR (95% CI)	2015^a^–2017,OR (95% CI)
Individual FeSS processes						
Fever treatment						
Paracetamol within 1 h^b, c^	192 (36)	186 (39)	226 (49)	1.1 (0.85, 1.5)	*1.7 (1.3, 2.2)*	*1.5 (1.1, 2.0)*
Overall fever treatment^b^	3099 (90)	3800 (93)	3952 (94)	*1.4 (1.2, 1.7)*	*1.7 (1.5, 2.1)*	*1.2 (1.0, 1.5)*
Hyperglycaemia treatment						
Insulin within 1 h^b^	160 (25)	204 (29)	256 (38)	1.3 (0.97, 1.6)	*2.0 (1.5, 2.5)*	*1.6 (1.2, 2.0)*
Overall hyperglycaemia treatment^b^	2947 (86)	3579 (88)	3781 (90)	*1.2 (1.0, 1.3)*	*1.5 (1.3, 1.8)*	*1.3 (1.1, 1.5)*
Swallow monitoring						
Swallow screen OR assessment within 24 h of hospital admission	2310 (62)	2618 (64)	2884 (69)	*1.2 (1.1, 1.3)*	*1.5 (1.3, 1.6)*	*1.3 (1.1, 1.4)*
Swallow screen or assessment before food or drink	1875 (50)	2222 (54)	2600 (62)	*1.2 (1.1, 1.4)*	*1.7 (1.6, 1.9)*	*1.4 (1.3, 1.5)*
Swallow screen or assessment before oral medications	1687 (45)	2047 (50)	2420 (58)	*1.3 (1.1, 1.4)*	*1.8 (1.6, 1.9)*	*1.4 (1.3, 1.5)*
Overall swallow monitoring	1401 (37)	1621 (40)	2059 (49)	*1.2 (1.1, 1.3)*	*1.8 (1.6, 1.9)*	*1.5 (1.4, 1.7)*
Swallow treatment						
Assessed by speech pathologist if failed swallow screen	562 (96)	769 (97)	826 (95)	1.3 (0.74, 2.3)	0.83 (0.50, 1.4)	0.64 (0.38, 1.1)
Swallow treatment^d^	3716 (99)	4061 (99)	4149 (99)	1.0 (0.58, 1.8)	0.62 (0.38, 1.1)	0.62 (0.38, 1.1)
Swallow monitoring and treatment	1387 (37)	1599 (39)	2027 (48)	*1.1 (1.0, 1.3)*	*1.7 (1.6, 1.9)*	*1.5 (1.4, 1.7)*
Composite outcome						
All elements of fever, sugar, swallow dysfunction monitored and treated	1024 (30)†	1290 (32)	1731 (41)	*1.1 (1.0, 1.2)*	*1.8 (1.6, 2.0)*	*1.6 (1.4, 1.8)*

Dependent variable in multivariable analyses is adherence to FeSS processes; independent variable is year, adjusted for correlation within hospital. Italicised results are significant

*FeSS* fever, sugar, swallow; *OR* odds ratio; *CI* confidence interval

^a^Year used as reference for multivariable analyses

^b^Excludes patients receiving palliative care in 2013

^c^In 2015/2017, those contraindicated to and already receiving regular paracetamol included as ‘no’ in denominator

^d^Assessed by speech pathologist if failed swallow screen—those who passed or did not receive swallow screen were considered to have received appropriate treatment

**Fig. 2:**
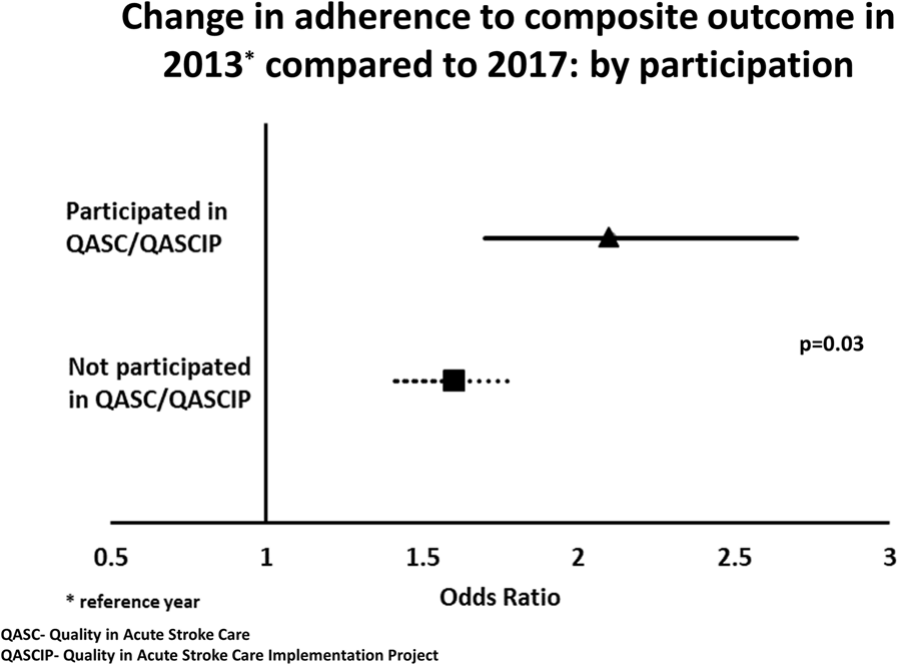
Change in adherence to the composite outcome in 2013 compared to 2017: by previous participation in QASC/QASCIP

In 2013, adherence to the composite outcome was similar for hospitals that had participated in QASC and those who had not (participated 31%, not participated 30%). The change in odds of adherence over time differed by participation (*p* < 0.001 for overall interaction term) and was generally larger for hospitals that had been involved with QASC/QASCIP compared to others in Australia (Table 4). The odds ratio of adherence for 2017 relative to 2013 was 2.1 (95% CI 1.7, 2.7) for hospitals that had previously participated and 1.6 (95% CI 1.4, 1.8; *p* = 0.03) for those that had not participated (Fig. 2). For comparable individual processes, adherence at hospitals that had previously participated in QASC/QASCIP completing the 2017 audit was found to be similar or improved against the QASCIP post-implementation cohort, particularly for fever and hyperglycaemia treatment processes (please see Additional file 2). Favourable improvements were also evident in hospitals in NSW or elsewhere in Australia that had not previously participated in QASC/QASCIP.

**Table 4:** Changes in adherence to composite outcome over time (2013–2017) by participation in QASC/QASCIP and the presence of a stroke unit

	2015 vs 2013^a^		2017 vs 2015^a^		2017 vs 2013^a^	
	OR (95% CI)	*p* value^b^	OR (95% CI)	*p* value^b^	OR (95% CI)	*p* value^b^
Participation in QASC/QASCIP						
Participated^c^	1.2 (0.95, 1.6)	0.4	1.7 (1.4, 2.0)	0.4	2.1 (1.7, 2.7)	0.03
Not participated^c^	1.1 (0.93, 1.2)		1.5 (1.4, 1.7)		1.6 (1.4, 1.8)	
Presence of a stroke unit						
Yes	1.0 (0.93, 1.2)	0.051	1.7 (1.5, 1.8)	0.09	1.7 (1.5, 1.9)	0.6
No	1.4 (1.04, 1.9)		1.4 (1.0, 1.8)		1.9 (1.4, 2.6)	

Dependent variable is adherence to composite outcome measure; independent variables include interaction term between year/participation QASC/QASCIP or year/stroke unit presence, adjusted for correlation within hospital. *OR* Odds Ratio, *CI* Confidence Interval

^a^Reference year

^b^For the interaction term

^c^Participated in QASC or QASCIP

**Fig. 3:**
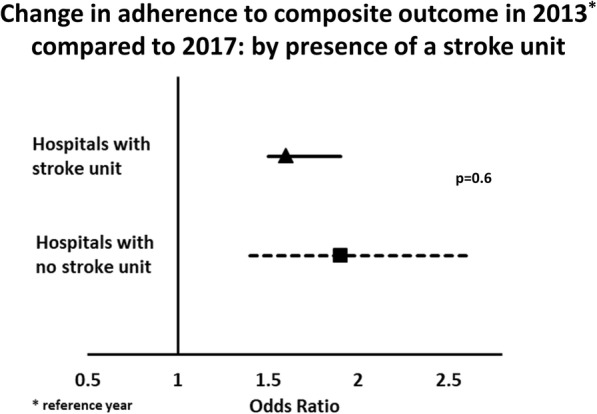
Change in adherence to the composite outcome in 2013 compared to 2017: by presence of a stroke unit

Changes in adherence to the composite outcome varied overall for hospitals with and without an SU (*p* < 0.001 for overall interaction term). Overall, there was no difference in odds of improvement evident between 2013 and 2017 (*p* = 0.6) (Fig. 3). However, adherence to the composite outcome in SU hospitals was still significantly greater than in those without an SU (2017—SU, 43%; non-SU 29%).

Results of the sensitivity analyses including patient characteristics and organisational factors in the multivariable modelling were consistent with our primary results (please see Additional files 3 and 4).

## Discussion

Results of our study show there has been increased uptake of the main FeSS processes across Australia over a 4-year period, with inclusion of these processes in the National Audit from 2013. Further, similar results were evident in both our primary and sensitivity analyses with adjustment for patient and organisational characteristics. These results are of particular interest in the wider context of stroke care in Australia, given that adherence to many other processes over the same time period has either stagnated or shown only minimal improvements [1]. These results are also relevant to other countries with similar health system resources to Australia, given the dearth of evidence-based nurse-led interventions for stroke [3], a leading cause of death and disability globally [25].

Translating evidence from clinical trials into routine clinical practice is inherently difficult [26], which can detrimentally affect patient care and outcomes [27]. Often, implementation activities are focused on short-term actions and effects [28]. Limited research has evaluated sustainability and uptake of evidence into practice post-implementation initiatives [29, 30]. Within the literature, various methods have been used to encourage routine adoption of evidence-based care, particularly related to care bundles, in clinical practice. In the review by Borgert and colleagues [15], it was demonstrated that audit and feedback was one of the most frequently used strategies to implement care bundles. Mandatory reporting [31], removal of perverse incentive payments [32] and more specific quality improvement programmes focused on combinations of building leadership, shared learning, mentoring and on-going measurement have also been used with effect [13, 33].

Adherence to the bundled care processes after the initial focused implementation efforts is often not maintained, or improved as in our study. Helmick and colleagues did report a small increase in compliance in ventilator-associated pneumonia and catheter-related bloodstream infection bundles after initial focused efforts to implement these into routine care [34]. Alternatively, Ferrer et al. reported that adherence to a sepsis care bundle returned to baseline 1 year after a national education programme was ceased [35]. This is also in line with a recent systematic review where adherence to guideline recommendations 1 year post-implementation with no further systematic implementation activities reduced in approximately 50% of studies [29]. However, sustained improvements in hospital care practices have been described from 6 to 36 months post-implementation in other conditions [36, 37]. Within stroke, there is variability. Sustained improvement in the provision of discharge care processes was evident 9 months post-implementation in one study [7]; however, in another multi-centre trial, improvements after the initial quality improvement effort were not sustained at 12 months [38]. Given the difficulties and variability in achieving knowledge translation and the considerable length of time it takes to embed evidence into standard practice [39], the results from the current study are notable indicating scale up and spread ‘at pace’ and provide insight into knowledge transfer in this context.

The use of ‘audit and feedback’ as an intervention in itself has been shown to be effective in improving healthcare delivery, with a median absolute improvement in the care of 4% (first quartile + 0.5%, third quartile + 16%) reported in a previous systematic review [40]. The data presented in our study represents two audit and feedback cycles of acute services. The feedback provided by the Stroke Foundation as part of the National Audit programme was relatively passive. No direction about implementing change was provided; rather, it was more around ‘monitoring’ care being delivered. This included generating and distributing a national report with aggregated data for individual care processes (e.g. not presented specifically as a FeSS care bundle) and individual site reports for all hospitals participating in each cycle. The overall net change of 10–14% improvement in many FeSS processes from our results compares favourably to the upper limits reported in previous studies of audit and feedback programmes [40]. These results highlight the potential benefits of integrating audit and feedback into exiting national registry and audit programmes. Consideration of how to actively provide feedback may help to consolidate these results further [41].

Adherence to the composite outcome in 2013 was similar in hospitals that participated in QASC and those that did not. Analyses were not conducted to examine differences by QASC group allocation to control or intervention, which could have diluted the effect of ‘participation’ in 2013. Overall, change in adherence to the composite outcome from 2013 to 2017 was greater in hospitals that had participated in the previous QASC/QASCIP interventions, where active dissemination of the FeSS processes occurred via workshops, protocols and the use of local clinical champions. In addition to the ‘audit and feedback’ implementation strategy of inclusion of related processes in the National Audit, other factors may have influenced the uptake further. These include publicity related to the original trial, conference presentations or publications of the QASC or QASCIP results. Additionally, the protocols and implementation strategies used in QASC were freely available to download online, both locally and internationally. While clinicians from 21 countries downloaded the resource, self-report of successful implementation resulting from this method alone was limited [42]. However, the lack of systematic data collected on the processes of care in different countries means we cannot objectively assess this.

In Australia, recent estimates indicate that only 75% of services providing acute stroke care have an SU [1]. Therefore, it was of interest to examine the care provided in hospitals without an SU related to the FeSS processes. While the absolute difference in adherence to the composite outcome between SU and non-SU hospitals is clear (2017 SU, 43%; non-SU, 29%), there was no difference in improved adherence between 2017 and 2013. Although it may be reasonable to assume that hospitals with an SU were already adhering to FeSS processes [43], improvements seen in non-SU hospitals may have also potentially been influenced by the lower baseline performance in non-SU hospitals [38]. In addition, the inclusion of the five non-SU NSW hospitals in the QASCIP study may have also affected these results. Although non-SU hospitals were few, there appears to be infiltration of the organisational processes involved in delivering these aspects of care more widely in all hospitals delivering acute stroke care.

Even considering the improvements in adherence to the composite outcome over time, a sizable evidence-practice gap remains. Only 41% received all FeSS processes (composite outcome) in 2017, with over half of those patients with fever and 3 in 5 with high glucose not receiving timely paracetamol and insulin, respectively. These findings are in line with a recent study where authors demonstrated sub-optimal fever and glucose management in stroke centres in the USA [44]. In addition, over a third of patients were still receiving oral medication/food/fluids prior to swallow screening, which is greater than that reported from recent data as part of the UK Sentinel Stroke National Audit Programme (26% did not get a swallow screen in 4 h) [16]. In view of the evidence demonstrating short and long-term improvement in patient outcomes associated with adherence to the FeSS processes [8, 10], an ongoing focus on reducing variation in care around these processes is necessary. Although the need for control of fever and hyperglycaemia have been made internationally [45], more stringent recommendations based on results of the QASC trial have since been included in national and international stroke guidelines [3, 46]. While adherence to some individual FeSS processes is measured in international audit programmes and registries [16, 17], to our knowledge, not all FeSS processes are captured in the same way as the original FeSS protocols outline. Therefore, it is difficult to generalise results related to the composite outcome to other countries. Other countries should be encouraged to collect data on FeSS process to provide important information on the effects of translation.

Strengths of the study include the large comprehensive nationally representative dataset and the use of a national data dictionary to reduce reporting bias and enhance the reliability of data collection. A limitation is that the composite outcome measure derived is different to that reported in the initial QASC trial [8], as not all the original FeSS monitoring processes were collected in subsequent audits. Changes primarily reflected efforts to reduce data burden for clinicians (please see Additional file 5). Even though direct comparisons with the composite measure are not possible, encouragingly, the adherence to other individual FeSS processes was found to be comparable or even improved against the QASCIP post-implementation cohort [6]. Limitations to the use of composite measures have been reported [47]. However, our methods to negate the influence of missing data with decision rules to ensure all patients were eligible to receive all processes in the measure and in-built logic checks in the data tool could address some of these concerns.

The aim of this study was not to provide an overview of improvements in adherence to the wider stroke evidence-base. In Australia, this is reported on biennially in the National Stroke Audit [1] and from the Australian Stroke Clinical Registry [2]. Rather, we chose to focus on uptake of the FeSS processes. This is an area not previously reported on, and related to a proven evidence-based nurse-initiated intervention. Investigating any association with changes in patient outcomes and improvements in FeSS processes was beyond the scope of this study, but an area of interest for future work.

Another limitation is the cross-sectional nature of a retrospective audit, with up to 40 cases, which only provides a snapshot of what is occurring over multiple audits and at each hospital. The audit data collection can also be influenced by documentation and responder bias. However, the web tool used for data entry ensured mandatory responses to questions, and the proportion of ‘not documented’ responses for process questions was similar over the audits, lending more confidence that the changes seen reflected improved care rather than improved documentation. Reliability checks involving repeated audits were performed to address this. Previous inter-reliability reports of indicators relating to the FeSS processes provided evidence of substantial agreement [6].

This study provides an example of the benefits of secondary use of data. As such, biases in retrospective data abstraction and results were not affected by prior knowledge of the study’s hypotheses. Inclusion of FeSS processes prior to 2013 was limited to swallow-related indicators that were not directly comparable to the processes included in the FeSS protocols. Therefore, no national baseline measure was available to determine potential secular trends in changes in adherences to these processes. Comparisons from 2013 to 2017 for some variables were also potentially influenced by additional changes to questions and responses between the 2013 and 2015 audit. Every effort was undertaken to ensure only comparable variables were included. Importantly, minimal changes were made between 2015 and 2017; therefore, these data provide a strong basis to assess adherence in future audits. The next acute audit in 2019 will provide important data to assess if there is a stronger uptake of the FeSS processes. While information related to swallow processes has been included in national guidelines since 2007 [48], recommendations related to the fever and hyperglycaemia FeSS protocols have now been included in the recently released 2017 national guidelines [3]. Therefore, the next audit will also provide stronger indirect evidence as to the impact of these new guideline recommendations.

## Conclusion

Increased adherence to the FeSS processes has occurred in Australia from 2013 to 2017 since the inclusion of these processes in the National Audit. Greater improvements were evident in hospitals where active exposure to the original intervention occurred. Further improvement in adherence to the FeSS processes is still required, but our implementation methods may be used in other translation initiatives, potentially beyond stoke care.

## Additional files

Additional file 1: Summary of baseline patient characteristics from National Audit periods. (DOCX 31 kb)

Additional file 2: Comparison of adherence to FeSS processes across QASC and QASCIP and hospitals completing 2017 National Audit (by previous participation in QASC/QASCIP). (DOCX 39 kb)

Additional file 3: Changes in adherence to FeSS processes and composite outcome over time - adjusted for patient and organisational factors. (DOCX 38 kb)

Additional file 4: Changes in adherence to composite outcome over time by participation and presence of a stroke unit - adjusted for patient and organisational factors. (DOCX 30 kb)

Additional file 5: Supplemental methods describing differences in data collection for FeSS processes over audit periods. (DOCX 19 kb)

## Data Availability

The data that support the findings of this study are available on reasonable request (from the Stroke Foundation at audit@strokefoundation.org.au).

## Abbreviations

CI: Confidence interval
FeSS: Fever, hyperglycaemia (sugar), swallow
NSW: New South Wales
QASC: Quality in acute stroke care
QASCIP: Quality in acute stroke care implementation project
SU: Stroke unit
