# Supplementary material for: Inclusion of a care bundle for fever, hyperglycaemia and swallow management in a National Audit for acute stroke: evidence of upscale and spread
Source: Implement Sci. 2019 Sep 2;14:87. doi: 10.1186/s13012-019-0934-y (PMC6721322; doi:10.1186/s13012-019-0934-y)
Supplement: Supplementary file 1 — Summary of baseline patient characteristics from National Audit periods. (DOCX 31 kb) [file 13012_2019_934_MOESM1_ESM.docx]

**Table I: Summary of baseline patient characteristics from National Audit periods**

|  | **2013 Audit**  **N=3,741**  **n (%)** | **2015 Audit**  **N=4,087**  **n (%)** | **2017 Audit**  **N=4,192**  **n (%)** | **p-value** |
| --- | --- | --- | --- | --- |
| **Patient characteristics** | |  |  |  |
| Male | 2,033 (54) | 2,244 (55) | 2,305 (55) | 0.83 |
| Median age (Q1,Q3) | 76 (65, 84) | 76 (65, 84)^a^ | 75 (65, 84) |  |
| Age group (years) |  |  |  |  |
| <65 | 928 (25) | 971 (24)^a^ | 1,011 (24) | 0.39 |
| 65-74 | 804 (21) | 927 (23)^a^ | 991 (24) |  |
| 75-84 | 1,147 (31) | 1,224 (30)^a^ | 1,250 (30) |  |
| ≥85 | 862 (23) | 957 (23)^a^ | 940 (22) |  |
| Independent prior stroke mRS 0-1 | 1,968 (58)^b^ | 2,801 (69) | 2,922 (70) | <0.001 |
| Diabetes | 938 (25) | 1,003 (25) | 1,010 (24) | 0.60 |
| Hypertension | 2,494 (67) | 2,675 (65) | 2,762 (66) | 0.52 |
| Hypercholesterolamia | 1,453 (39) | 1,514 (37) | 1,611 (38) | 0.22 |
| Ischaemic heart disease | 928 (25) | 1,008 (25) | 973 (23) | 0.18 |
| Previous stroke or TIA | 1,069 (29) | 1,264 (31) | 1,207 (29) | 0.04 |
| **Stroke type** |  |  |  |  |
| Ischaemic stroke | 2,872 (77) | 3,121 (76) | 3,353 (80) | <0.001 |
| **Stroke severity on admission**^c^ | |  |  |  |
| Arm weakness | 2,430 (65) | 2,406 (59) | 2,418 (58) | <0.001 |
| Impaired speech | 2,099 (56) | 2,297 (56) | 2,306 (55) | 0.48 |
| Unable to walk | 2,508 (67) | 2,197 (54) | 2,173 (52) | <0.001 |
| Incontinence at 72 hours | 1,352 (36) | 1,359 (33) | 1,387 (33) | 0.006 |

Q1- 1^st^ quartile; Q3- 3^rd^ quartile; TIA- transient ischaemic attack; mRS- modified Rankin Score; ^a^<1% missing data; ^b^missing 8% data; ^c^patients receiving palliative care in 2013 recorded as having all severity measures.
