# Supplementary material for: Inclusion of a care bundle for fever, hyperglycaemia and swallow management in a National Audit for acute stroke: evidence of upscale and spread
Source: Implement Sci. 2019 Sep 2;14:87. doi: 10.1186/s13012-019-0934-y (PMC6721322; doi:10.1186/s13012-019-0934-y)
Supplement: Supplementary file 2 — Comparison of adherence to FeSS processes across QASC and QASCIP and hospitals completing 2017 National Audit (by previous participation in QASC/QASCIP). (DOCX 39 kb) [file 13012_2019_934_MOESM2_ESM.docx]

**Table II: Comparison of adherence to FeSS processes across QASC and QASCIP and hospitals completing 2017 National Audit (by previous participation in QASC/QASCIP)**

|  |  | **QASC post intervention**  **2010 N=603 n (%)** | **QASCIP post evaluation**  **2014 N=1,082 n (%)** | **National Audit 2017** | |
| --- | --- | --- | --- | --- | --- |
| **Element** | **Comparable monitoring and treatment processes based on FeSS Protocols from QASC^a^** |  |  | **Participated in QASC/QASCIP**  **N=1,495 n (%)** | **Did not participate in QASC/QASCIP**  **N=2,697 n (%)** |
|  |  |  |  |  |  |
|  | FEVER TREATMENT |  |  |  |  |
|  | Paracetamol within 1 hour for first elevated fever^b^ | 19 (18) | 64 (47) | 66 (64) | 160 (44) |
|  | HYPERGLYCAEMIA TREAMENT |  |  |  |  |
|  | Insulin within 1 hour for first elevated finger-prick glucose | 19 (14) | 56 (27) | 104 (53) | 152 (32) |
|  | SWALLOW MONITORING (M) and TREATMENT (T) |  |  |  |  |
| M1 | Received a swallow screen OR swallow assessment within 24 hours of hospital admission | 491 (81) | 814 (75) | 922 (72) | 1,962 (67) |
| M2 | Received a swallow screen or assessment before food or drink | 135 (22) | 736 (68) | 846 (66) | 1,754 (60) |
| M3 | Received a swallow screen or assessment before oral medications | 222 (37) | 670 (62) | 799 (62) | 1,621 (56) |
|  | Treatment according to protocol for monitoring swallow dysfunction (if M1, M2 and M3 ALL met) | 65 (11) | 565 (52) | 691 (54) | 1,368 (47) |
| T1 | Failed swallow screen and received a swallow assessment by a speech pathologist | 74 (78) | 218 (95) | 300 (95) | 526 (95) |
|  | Monitored and treated according to protocol for swallowing dysfunction (if M1, M2, M3 and T1 ALL met) | 62 (10) | 556 (51) | 678 (53) | 1,349 (46) |

FeSS: fever, sugar, swallow; QASC: Quality in Acute Stroke Care; QASCIP: Quality in Acute Stroke Care Implementation Project; ^a^ Monitoring components for fever and hyperglycaemia not available in the audit data; ^b^2017 excludes those contraindicated to and already receiving regular Paracetamol
