# Supplementary material for: Inclusion of a care bundle for fever, hyperglycaemia and swallow management in a National Audit for acute stroke: evidence of upscale and spread
Source: Implement Sci. 2019 Sep 2;14:87. doi: 10.1186/s13012-019-0934-y (PMC6721322; doi:10.1186/s13012-019-0934-y)
Supplement: Supplementary file 3 — Changes in adherence to FeSS processes and composite outcome over time - adjusted for patient and organisational factors. (DOCX 38 kb) [file 13012_2019_934_MOESM3_ESM.docx]

**Table III: National changes in adherence to FeSS processes and composite outcome over time (2013-2017) adjusted for patient characteristics and organisational factors**

| **FeSS monitoring and treatment processes** | **Multivariable**^a^ | | |
| --- | --- | --- | --- |
|  | **2013** | **2015**  **OR (95% CI)** | **2017**  **OR (95% CI)** |
| ***INDIVIDUAL FeSS PROCESSES*** |  |  |  |
| ***Fever treatment*** |  |  |  |
| Paracetamol within 1 hour^b,c^ | 1.0 | 0.99 (0.74, 1.3) | **1.5 (1.1, 2.0)** |
| Overall fever treatment^b^ | 1.0 | **1.3 (1.1, 1.5)** | **1.5 (1.3, 1.9)** |
| ***Hyperglycaemia treatment*** |  |  |  |
| Insulin within 1 hour^b^ | 1.0 | 1.3 (0.95, 1.7) | **2.0 (1.5, 2.6)** |
| Overall hyperglycaemia treatment^b^ | 1.0 | 1.1 (0.98, 1.3) | **1.5 (1.3, 1.7)** |
| ***Swallow monitoring*** |  |  |  |
| Swallow screen OR assessment within 24 hours of hospital admission | 1.0 | **1.1 (1.00, 1.3)** | **1.3 (1.2, 1.5)** |
| Swallow screen or assessment before food or drink | 1.0 | **1.3 (1.2, 1.4)** | **1.8 (1.6, 2.0)** |
| Swallow screen or assessment before oral medications | 1.0 | **1.3 (1.2, 1.5)** | **1.9 (1.9, 2.1)** |
| Overall swallow monitoring | 1.0 | **1.2 (1.1, 1.3)** | **1.8 (1.6, 2.0)** |
| ***Swallow treatment*** |  |  |  |
| Assessed by speech if failed swallow screen | 1.0 | 1.2 (0.67, 2.3) | 0.77 (0.43, 1.4) |
| Swallow treatment§ | 1.0 | 0.87 (0.48, 1.6) | **0.51 (0.29, 0.89)** |
| ***Swallow monitoring and treatment*** | 1.0 | **1.2 (1.0, 1.3)** | **1.7 (1.6, 1.9)** |
| ***COMPOSITE OUTCOME*** |  |  |  |
| ALL elements of fever, sugar, swallow dysfunction monitored and treated | 1.0 | 1.1 (0.99, 1.2) | **1.7 (1.5, 1.9)** |

FeSS- fever, sugar, swallow; ^a^2013 used as reference year for all multivariable analyses. Dependent variable is adherence to FeSS processes, independent variables includes year, age, sex, pre-morbid independence, stroke severity including arm weakness, ability to walk on admission and incontinence within 72 hours, ischemic stroke, use of protocols to manage fever, hyperglycaemia and swallow, presence of a SU, adjusted for correlation within hospital; ^b^excludes patients receiving palliative care in 2013; ^c^In 2015/2017 those contraindicated to and already receiving regular Paracetamol included as ‘no’ in denominator; §assessed by speech pathologist if failed swallow screen- those who passed or did not receive swallow screen were considered to have received appropriate treatment; OR: odds ratio; CI; Confidence interval; **Bold Results** are significant
