# Supplementary material for: Inclusion of a care bundle for fever, hyperglycaemia and swallow management in a National Audit for acute stroke: evidence of upscale and spread
Source: Implement Sci. 2019 Sep 2;14:87. doi: 10.1186/s13012-019-0934-y (PMC6721322; doi:10.1186/s13012-019-0934-y)
Supplement: Supplementary file 4 — Changes in adherence to composite outcome over time by participation and presence of a stroke unit - adjusted for patient and organisational factors. (DOCX 30 kb) [file 13012_2019_934_MOESM4_ESM.docx]

**Table IV: Changes in adherence to composite measure over time (2013-2017) by participation in QASC/QASCIP and presence of a stroke unit - adjusted for patient characteristics and organisational factors**

|  | **2015 vs 2013^a^** | | **2017 vs 2015^a^** | | **2017 vs 2013^a^** | |
| --- | --- | --- | --- | --- | --- | --- |
|  | **OR (95% CI)** | **p-value**^b^ | **OR (95% CI)** | **p-value**^b^ | **OR (95% CI)** | **p-value**^b^ |
| **Participation in QASC/QASCIP** | | |  |  |  |  |
| Participated^c^ | 1.1 (0.87, 1.5) | 0.1 | 1.7 (1.4, 2.0) | <0.001 | 1.9 (1.5, 2.4) | 0.007 |
| Not participated^c^ | 1.0 (0.90, 1.2) |  | 1.5 (1.3, 1.7) |  | 1.5 (1.4, 1.8) |  |
| **Presence of a stroke unit** | |  |  |  |  |  |
| Yes | 1.1 (0.93, 1.2) | 0.09 | 1.6 (1.5, 1.8) | 0.052 | 1.7 (1.5, 1.9) | 0.6 |
| No | 1.6 (1.1, 2.2) |  | 1.4 (0.98, 1.9) |  | 2.2 (1.5, 3.2) |  |

OR: odds ratio; CI: confidence interval; ^a^reference year; ^b^for the interaction term; ^c^Participated in QASC or QASCIP. Dependent variable is adherence to composite outcome measure, independent variables include interaction term between year/participation or year/stroke unit presence, age, sex, pre-morbid independence, stroke severity including arm weakness, ability to walk on admission and incontinence within 72 hours, ischemic stroke, use of protocols to manage fever, hyperglycaemia and swallow, presence of a stroke unit (for participation), adjusted for correlation within hospital.
