# Supplementary material for: Inclusion of a care bundle for fever, hyperglycaemia and swallow management in a National Audit for acute stroke: evidence of upscale and spread
Source: Implement Sci. 2019 Sep 2;14:87. doi: 10.1186/s13012-019-0934-y (PMC6721322; doi:10.1186/s13012-019-0934-y)
Supplement: Supplementary file 5 — Supplemental methods describing differences in data collection for FeSS processes over audit periods. (DOCX 19 kb) [file 13012_2019_934_MOESM5_ESM.docx]

**Supplemental methods describing differences in data collection for FeSS processes over audit periods**

Between the 2013 and 2015 audit, some changes to the questions were made. To minimise data collection burden for clinicians, questions relating to regular monitoring of fever and glucose levels in the first 72 hours post arrival were removed. Other changes related to response options. For example, ‘administration of paracetamol for the first elevated temperature’, changed from ‘yes/no/not documented’ in 2013, to include ‘already on regular paracetamol’ and ‘contraindicated’ as well from 2015 onwards. For consistent comparison, the ‘already on regular paracetamol’ and ‘contraindicated’ responses were recorded as ‘no’ and included in the denominator in the 2015 and 2017 analyses.

In addition, in 2013, in-built logic programmed within the web-tool for the cohort of patients receiving palliative care (8%), meant many questions relating to impairments on admission and processes of care were not answered for these cases. As a consequence, in 2013 only, for questions relating to the severity of stroke based on impairments reported on admission (arm weakness, inability to walk, speech impairment and incontinence), responses were assumed to be positive for patients who were palliated. However, for processes of care, missing responses for patients who were palliated were excluded from the 2013 dataset for all analyses.
